# Making decisions about antipsychotics: a qualitative study of patient experience and the development of a decision aid

**DOI:** 10.1186/s12888-019-2304-3

**Published:** 2019-10-23

**Authors:** S. J. Kaar, C. Gobjila, E. Butler, C. Henderson, O. D. Howes

**Affiliations:** 1 Department of Psychosis Studies, Institute of Psychiatry, Psychology and Neuroscience (IoPPN), King’s College London, PO63 De Crespigny Park, London, SE5 8AF UK; 2 Health Service and Population Research, Psychological and Systems Sciences, Institute of Psychiatry, Psychology and Neuroscience (IoPPN), King’s College London, London, SE5 8AZ UK; 3 Psychiatric Imaging Group, MRC London Institute of Medical Sciences, Imperial College, Hammersmith Hospital, London, UK

**Keywords:** Shared decision making, Adverse effects, Informed consent, Antipsychotic, Patient preference

## Abstract

**Background:**

Shared decision making is a widely accepted standard of patient-centred care that leads to improved clinical outcomes, yet it is commonly underutilised in the field of mental health. Furthermore, little is known regarding patient decision making around antipsychotic medication, which is often poorly adhered to. We aim to explore psychiatric patients’ experiences of antipsychotic medication decision making in order to develop a patient decision aid to promote shared decision making.

**Methods:**

Focus groups were conducted with patients with chronic psychotic illnesses (*n* = 20) who had previously made a decision about taking or changing antipsychotic medication. Transcripts were coded and analysed for thematic content and continued until thematic saturation. These themes subsequently informed the development of a decision aid with the help of expert guidance. Further patient input was sought using the think aloud method (*n* = 3).

**Results:**

Twenty-three patients participated in the study. Thematic analysis revealed that ‘adverse effects’ was the most common theme identified by patients surrounding antipsychotic medication decision-making followed by ‘mode and time of administration’, ‘symptom control’ and ‘autonomy’. The final decision aid is included to provoke further discussion and development of such aids.

**Conclusions:**

Patients commonly report negative experiences of antipsychotic medication, in particular side-effects, which remain critical to future decision making around antipsychotic medication. Clinical encounters that increase patient knowledge and maximise autonomy in order to prevent early negative experiences with antipsychotic medication are likely to be beneficial.

## Background

Shared decision making is a widely accepted standard of patient-centred care [1] where both the patient and clinician contribute to a medical decision by sharing information and responsibility [2]. It is a process within a clinical encounter that involves a clinician explaining the available treatment options, the patient thinking about and discussing their values and preferences with the clinician, before ascertaining their priorities through reflection and information gathering, then finally, both parties coming to a shared decision about the treatment that best aligns with the unique beliefs of the patient [3, 4].

Recent systematic reviews show that patients who actively participate in shared decision making have higher satisfaction with their care [5, 6] and less decisional conflict characterised by uncertainty about the best course of action among competing options involving risk, regret, or challenge to personal values [7]. Despite shared decision making being a widely accepted standard of patient-centred care [1], it is commonly underutilised in the field of mental health [8]. Involving patients more closely in the decisions about psychiatric medication has been suggested as a means of combating high levels of non-adherence and reducing excessive prescribing [9]. A meta-analysis of randomised control trials of shared decision making for treatment of psychosis found a small benefit effect on indices of treatment-related empowerment but noted that the six included studies were of low quality and the effect size small (Hedges *g* = 0.3) [10]. Two studies within mental health hospital settings found that shared decision making was associated with positive experiences of care [11, 12], and a systematic review found that authentic experiences of patient centred care, of which shared decision making is a part of, was a key theme in the inpatient experience [13]. In the community setting a shared decision making training programme was able to significantly change patients’ decisional conflict [14]. However, one trial of an antipsychotic decision aid on psychiatric inpatients showed positive short term effects in patients’ perceived involvement in decision making [15], but subsequently failed to show a significant difference in longer term outcomes (rehospitalisation and medication adherence) [16].

Antipsychotic medication has been shown to effectively reduce the symptoms of psychosis through dopamine receptor blockade [17], although actions at other receptors may also contribute to efficacy [18]. Antipsychotic use is associated with a range of adverse effects that are often difficult to manage [19] and can lead to secondary negative and cognitive symptoms [20, 21]. Such adverse effects contribute to treatment non-adherence [22] and poorer outcomes [23, 24]. Balancing the benefits of pharmacological interventions against resultant negative effects is complex given that adverse effects can significantly impair patients’ quality of life [25]. Notwithstanding the effects of medication, psychotic disorders impair cognitive function [26] and affect insight [27], which may further complicate decision-making about treatment. For these reasons, making a decision about antipsychotic treatment is complex and can be challenging for patients, carers and clinicians. Much remains to be determined about how patients with psychotic illnesses make decisions regarding antipsychotic medication, although some work has been conducted in women who made decisions about antipsychotics or mood stabilisers during pregnancy [28, 29]. Peterson [28] found that information seeking, experiential evidence and access to services were critical to decision making. Pinfold [29] found that women seek to maintain personal control over decisions whilst also recognising the constraints brought about by mental health needs. Furthermore, whilst patients with psychotic disorders value the positive effects of medication, many report coercion and a lack of equality in decision-making [30]. A report by the charity Rethink [31] supports such findings, as many patients reported limited information giving and choice when an antipsychotic prescribing decision was made.

Decision aids are preference-based tools that help patients to understand the key areas of information relevant to a decision in a non-persuasive manner [32], and so can be used to formalise a shared-decision making process [33]. Yet only one group has continued to develop a decision aid for antipsychotic medication in psychosis [34], however, this study did not explicitly explore patient’s experiences of decision making around antipsychotic medication. In view of this, we undertook a qualitative study to explore the experiences of psychiatric patients within the context of antipsychotic decision making. This information, along with expert guidance, was used to develop a decision aid designed to systematise a patient’s values and preferences around medication, in order to encourage shared decision making in clinical encounters involving a change in antipsychotic medication.

## Methods

A qualitative design was used to gain insight into the personal beliefs and attitudes of patients towards antipsychotic medication, and the processes employed by patients in making decisions regarding their medication.

### Sample characteristics

Participants were invited to join the study if they were current mental health patients within the South London and Maudsley NHS Foundation Trust, had a history of a psychotic disorder meeting ICD-10 criteria [35], and were taking an antipsychotic treatment. Participants were recruited by purposeful sampling [36] through local mental health teams. Written informed consent was obtained prior to inclusion in the study. Participants also agreed to have the focus groups transcribed and for anonymous quotations to be used for publication. See Additional file 1 for more detailed patient characteristics.

The study was approved National Research Ethics Service Committee London – Brent (REC reference: 13/LO/0538). Focus groups were conducted in 2017.

### Data collection

Focus groups (*n* = 4, range 3–6 patients) lasted between 60 and 90 min. Both researchers and participants had no prior interaction. The focus groups were conducted by CG and SK. Open questions were asked about the factors that influenced a decision to take an antipsychotic treatment. Topics covered included the benefits, adverse effects, methods of administration, physical health monitoring and who was involved in the decision-making process. The emotional aspects of these experiences were enquired about as well as practical aspects, and focus groups allowed flexibility for participants to discuss emerging themes in detail. Participants were asked to reflect upon their experiences with antipsychotic medication, and comment specifically on anything they wished they had known before making the decision to start their current or past antipsychotic treatment. They were also asked to comment on their expectations of antipsychotic treatment, problems with medication they would like to avoid, and feelings about their relationships with their prescriber. A topic guide (see Additional file 1) was used during each focus group however researchers ensured questions remained broad and open to promote the emergence of new themes. All participants were encouraged to contribute during the discussion. The aim of the focus groups was to obtain adequate information about the values, preferences and priorities of patients when making decisions about antipsychotic medication, to inform the development of a medication decision aid.

### Data analysis

The focus groups were recorded and transcribed verbatim, and fully analysed by employing thematic analysis [37]. The transcripts were separately analysed, and phrases were then coded in the initial round of analysis. An inductive approach was used to analyse the data [38]. Common attitudes and experiences, referred to as ‘themes’, were identified in the transcripts and coded. The themes were then reviewed and discussed by the research team to establish relevance. The software QSR NVivo version 11 for Windows [39] was used to code and analyse the transcripts. The software determined the frequency with which themes were raised across focus groups. A framework of specific codes was developed to describe participant experiences with antipsychotic medication, and, more generally, with psychiatric services over time, until theoretical saturation was reached [40] and no new themes were apparent.

### Development of the decision aid

Following the focus groups a draft decision aid was developed based on the themes and issues raised in the focus groups (see Additional file 2). Expert guidance was sought through consultation within the multidisciplinary team in the Maudsley Treatment and Review Service, South London and Maudsley NHS Trust, UK, which specialises in the assessment and treatment of complex and treatment resistance cases of psychosis, and contributes to the Maudsley Prescribing Guidelines [41, 42], to ensure that a wide range of positive and adverse medication effects were captured within the decision aid. Think aloud sessions (*n* = 3) were conducted by EB and SK to provide the researchers with feedback on the draft decision aid. One participant had taken part in one of the focus groups, and the two others had not. The two patients who had not previously taken part were recruited from a pool of patients who have consented to be contacted for research within the South London and Maudsley NHS Trust. Think aloud sessions are a frequently used method of evaluating the usability of interventions [43, 44]. Unlike focus groups think aloud sessions are conducted in a one to one setting that allows a single participant to provide verbal responses whilst engaging in an intervention. During a one to one meeting with a researcher the participant was asked to use and think about a draft of the decision aid and encouraged to verbalise their thinking. Think aloud sessions were recorded for accuracy and the decision aid was revised after each session using an iterative approach until saturation was reached. Examples of think aloud feedback are included in the Additional file 1.

## Results

We conducted focus groups (*n* = 4) with 20 patients in total of which 67% were male, the average age was 43 years old, 62% were non-white and the most common diagnosis was schizophrenia (85%). The mean time under the care of mental health services was 19 years and each patient was taking at least one antipsychotic medication (mean 1.2) (for patient demographics and clinical characteristics see Table 1.). There were four main recurrent themes identified across transcripts: adverse effects were discussed by all but two participants, making it the primary and most common theme across transcripts, mode and time of administration was discussed by half of the participants, followed by symptom control and autonomy.

**Table 1:** Participants’ demographic and clinical characteristics

Participants (*n* = 23)	
Age, years: mean (s.d.) range	43 (10) 25–58
Male: *n* (%)	16 (70)
Employment, *n* (%)	
Employed, full/part-time	3 (13)
Unemployed/DLA or incapacity benefit	20 (87)
Ethnicity, *n* (%)	
British, White	11 (48)
British, Carribean	7 (30)
British, mixed	1 (5)
Other	4 (17)
Location of recruitment, *n* (%)	
Community mental health teams	22 (96)
Specialist mood disorders clinic	1 (4)
Diagnosis received, *n* (%)	
Schizophrenia	20 (87)
Schizoaffective disorder	2 (8)
Bipolar II disorder	1 (4)
Medication Taken, *n* (%)	
Clozapine	11 (47)
Paliperidone	4 (17)
Risperidone	3 (13)
Aripiprazole	2 (9)
Olanzapine	1 (4)
Quetiapine	1 (4)
Chlorpromazine	1 (4)
Amisulpride	1 (4)
Citalopram	2 (8)
Metformin	1 (4)
Bendroflumethiazide	1 (4)
Procyclidine	1 (4)
Atorvastatin	1 (4)
Zopiclone	1 (4)
Clonazepam	1 (4)

### Theme 1: adverse effects of antipsychotic medication

Adverse effects and their likelihood were the primary topic of discussion during the focus groups and appeared to be the overriding factor when patients thought about decision making around changing antipsychotic. All patients reported at least some degree of lethargy associated with either current or past antipsychotic medication.

‘It made me want to sleep, but at the same time it made me want to just die. Is this what life is all about? I couldn’t get out much... I was just lying on my back all the time, and it wasn’t natural, it didn’t feel natural. It just felt like I was under something, like somebody has got me in chains.’ (P01)

Problems with swallowing and excessive saliva production were also among the commonly reported issues described by the majority of participants.

‘You are sleeping, and you wake up, and it’s [saliva] on the pillow and you’re coughing.’ (P03)

Half of the participants described problems with increased appetite and unwanted weight gain.

‘Sometimes I take medication and go to sleep, and I feel hungry in the night, so I will wake up and eat something.’ (P16)

Some disclosed problems with balance and unsteadiness on their feet.

‘I started having dizzy spells and blackouts and that went on for about a year and a half until I pinpointed it down to the medication and changed it.’ (P13)

Some reported issues with memory, directly attributed to taking antipsychotic medication.

‘My tablets make me forget everything!’ (P09)

To a lesser degree, patients described loss of appetite, altered sleeping patterns, restlessness, and dysarthria.

### Theme 2: mode and time of administration of medication

There were many accounts highlighting the importance of the way a medication is administered, with particular emphasis on patient preference. The discussion revolved around oral versus injectable antipsychotics, their efficacy, and whether the mode of administration had an impact on satisfaction with treatment and long-term adherence.

Some patients described ‘being in charge’ as an important aspect of dealing with their illness. Oral medication provided a degree of freedom and self-control, offering the necessary coping mechanisms deemed desirable by the majority of participants.able by the majority of participants.

‘Being with self-medicated gives you a little bit more confidence because you are in control of it yourself.’ (P05)

A number of patients favoured injectable (depot) medication due to the reduced number of medical visits and the reduced risk of non-adherence.

‘The injection I only have to once every three months … which I find is perfect, even remembering it you think you’d forget it it’s so long but you remember as each week gets nearer.’ (P18)

Some participants agreed that the major problem with injectable medication was the injection itself. They found the physical contact with the needle often painful, discouraging them from considering injectable medication.

‘I could not stand injections because of the lump that it makes and this is just terrible. You get the pain from the spot where they inject you, you get a lump … ’ (P07)

Some felt strongly against injectable medication on account of stigma surrounding their illness. It trumped all possible benefits and deterred them from ever considering injectable medication as a viable option.

‘Needles used to make me feel shame, I used to feel embarrassed taking them. It felt like people are watching you take the needle, and then you take the needle, and then they laugh about that.’ (P01)

### Theme 3: symptom control

The benefits patients experienced in the past with medication helped to guide their decision making. Patients offered detailed insight into the aspects of their illness tackled by antipsychotic medication, as well as the long-term benefits in terms of quality of life improvement.

Most patients deemed the sedating effect experienced with antipsychotic to be highly desirable.

‘I take Quetiapine but I also take Chlorpromazine if I’m not feeling well and I had a lot yesterday and it made me feel like you said very like a zombie but in a way it was good because it’s what I needed yesterday.’ (P09)

Those who have previously taken clozapine or were taking clozapine at the time of the study, noticed a marked improvement in terms of sleep and auditory hallucinations, which, in turn, had a positive effect on the rest of their symptoms.

‘ … now with clozapine, no problem at all. The voices have gone down, no problems with sleep. My sleeping is slowly increasing, so I have no problem at all.’ (P02)

A few patients noticed some improvement of the paranoia associated with their illness.

‘I feel less paranoid taking the medicine I’m taking now. I’m more mentally aware myself.’ (P10)

In addition to positive effects from medication in the past, expectations of future positive effects of a medication also guided patient’s decision making. The discussion primarily focused on symptoms of illness. Specifically, patients voiced their concerns with regard to auditory hallucinations.

‘ … the … psychiatrist said “Well, it’s 97% of stopping your voices”, and, once you hear that, your brain goes like “Great, I’ll take it!” All you think that is it is great that there will not be voices … ’ (P04)

Again, the focus shifted from benefits of antipsychotics to dealing with some of their adverse effects. The majority were concerned about the sedative effect of medication, and how it prevented patients from carrying on with their daily lives.

‘I just feel like at the beginning it was like you’ve got to take this, this will make you better. They didn’t tell me the long run like the long run that it gets more and more, you get more drowsy. When one medication wasn’t working, oh this is a better one, and then it was this is a better more and now I’m on this and I don’t know where I’m coming from one day to the next.’ (P08)

It was unanimously agreed upon that anything that helps them feel well again is highly attractive.

‘Just to be well all the time makes me happy. Just to feel normal again and not sick and unwell and overtired and hallucinating.’ (P12)

### Theme 4: autonomy and decision making

Patients voiced concerns with regard to disempowerment in the decision-making process due to improperly presented information.

‘I was … trying to understand what the doctors are saying, because you walk into a doctors surgery at best of times, and they start talking about this medication, all this medical stuff, and you’re sitting there thinking “What are they saying?...”’ (P02)

The lack of information often precipitated distress and anger towards doctors and substantially affected adherence to medication.

‘They came and gave a little tub of something, a liquid, and said “Here, take this, this is going to help you!” So I’m like “Wait, how will help me?” What do you mean “it is going to help me”?! What is it?! It’s weird, isn’t it? Of course, I would not be ok.’ (P01)

Part of the debate revolved around what was perceived as actually helpful. Some employed all available resources to find out more about their illness, medication, and what to expect from mental health services.

‘ … wanting to know more about your illness and your medication if you’ve got the option. I do go to the library a lot and read a lot of books and the causes of schizophrenia. It’s quite interesting to read because … when you read some … it’s like you are reading about yourself.’ (P10)

Others found this kind of information neither helpful nor engaging. In these cases, the doctor’s professional expertise was valued above all else, and the rapport built over time trumped any external input.

‘Well, all the medication that I get, it comes with boxes and they’ve got this little pamphlet with something written on it. I have never read any of them, and every box has one.’ (P05)

Some patients made reference to early past experiences with mental health services.

‘ … I started off on fluoxetine and olanzapine first of all, and these were having mild effects. And then, I started with depression first, and then psychosis hit afterward. You start getting your medication, and they’re upping it, and upping it, and upping it, and they see a nice baseline, as they call it. And, I then changed teams, and bam, you’ve been on this drug, it doesn’t seem to have worked, so then they throw another one at you, and that doesn’t seem to work, so they throw another one at you. And you’re like “why so many pills all the time?”, and you’re like “have I done something wrong?”’ (P03)

The desire to reduce or altogether stop medication was voiced by a minority of the patients.

‘May be in the future I would like to come off medication and be free of medication.’ (P18)

### Decision aid development

To address the key issues raised by the patient focus groups, we developed a decision aid tool using think aloud sessions. The decision aid (see Additional file 1) aims to crystallise the key areas of information on individual priorities and past experiences to help patients make a decision about medication. It includes prompts to encourage reflection on past experiences with medication and space for the documentation of values, preferences and prioritisation of potential benefits and adverse effects associated with medication. Through sections on adverse effects, mode and time of administration and symptom control it covers three of the themes identified in the focus groups. The final focus group theme of autonomy and decision making is addressed by the tool providing documentary evidence of a patient’s individual values and preferences that can be taken into a clinical encounter to aid the share decision making process. To further develop the decision aid, we conducted three think aloud sessions with two participants who had not participated in the focus groups and one who had. Participants identified the need to provide a better explanation of the purpose of the decision aid, to simplify and improve the accuracy of the language used and use a rating scale that encouraged better prioritisation of needs.

## Discussion

Our analysis of patients’ opinions and beliefs revealed that the driving factor in reaching a decision regarding an antipsychotic medication revolves primarily around the adverse effect profile of the medication in question. Patients often focused on negative experiences of medication. Positive experiences also seem to be a contributing factor in decision making, though to a lesser extent. This is consistent with findings by Izadi and colleagues [45] which suggest that patient decision making may be driven by both affective processes and arguments presented by the psychiatrist, notwithstanding the fact that patients also seek information on the positive and negative effects of antipsychotic medication from multiple sources, in order to arrive at a decision [30]. Our study found that negative experiences with medication were more prominent among responses than positive ones, and these experiences tend to stay with the patient over many years and affect future decision making. However, as our study was not quantitative and the sample may not be representative of patients in general, this observation requires testing. This highlights the need for psychiatrists to take a detailed medication history with a focus on past experiences of medication and to be explicit in their discussion with patients about the benefits and adverse effects of an antipsychotic and, whenever possible, have a conversation about the comparative risks and benefits of different antipsychotic options [46].

The way a medication is administered was an important consideration. Interestingly participants were divided between injectable/depot and oral medication. This variation suggests that it is important not to make assumptions about patient preferences on the mode of administration and to have an early discussion on this topic. Survey data shows that patients have differing levels of acceptance of depot medication, with those currently or previously prescribed a depot more likely to accept this form of administration [47]. This further highlights the important of past experiences in future medication decisions.

Our study demonstrates the complexity of making decisions about antipsychotic treatment. Decision aids have been suggested as a means of simplifying this process and promoting patient involvement [48]. A decision aid is a tool designed to facilitate shared decision making and actively encourage patient participation in healthcare decisions [49]. A recent Cochrane review of decision aids concluded that people exposed to such aids report to be better informed and have a more active role in decision making [50]. Trial data suggests that decision aids improve patients’ knowledge of risks and benefits [51] and reduce decisional conflict [52]. In depression, decision aids have been demonstrated to encourage a more active role in decision-making [53] and improved decisional comfort, knowledge and satisfaction, despite failing to improve medication adherence [54]. Zisman-Ilani and colleagues [34] found that their decision aid was valuable and acceptable to patients changing their antipsychotic, who felt better able to raise concerns and questions.

### Limitations

Some limitations of this study need to be addressed. The findings might have been influenced by researcher biases. However, focus groups were undertaken by a non-clinician post-graduate neuroscience researcher (CG) and a clinical academic psychiatrist, (SK). Care was taken to avoid influencing the focus groups discussion. Similarly, the think aloud sessions were conducted by SK and EB, a non-clinician post-graduate neuroscience researcher, who was not involved in designing the first iteration of the decision aid. The sample size is relatively small, although is comparable to similar qualitative studies [55, 56]. Moreover, we achieved theoretical saturation, indicating that the main themes are stable. In addition, the think aloud method is designed to obtain in-depth data from small sample sizes and our n (*n* = 3) is fairly typical [57, 58]. Finally, our cohort was recruited from local services in South London and may not necessarily be representative of other areas and services.

Whilst we sought expert advice on medication effects to consider, additional input from a wider range of experts, including user experts, using a Delphi method could be used to refine the aid. Additionally, it would be useful to conduct a further qualitative study in a larger, diverse sample. Further studies will be required to develop, and test models of shared decision making based on the themes we identify. The next key step is to formally test the efficacy of our decision aid in improving decision making for patients and on clinical outcomes such as adherence in a pilot study.

## Conclusions

Our data suggests that being actively involved in decision making about antipsychotic treatment is important to patients, and the discussion should consider adverse effects, mode of administration and symptom relief. Clinical encounters that increase patient knowledge and maximise autonomy in order to prevent early negative experiences with antipsychotic medication are likely to be beneficial. Future research is necessary to determine the feasibility and validity of an antipsychotic decision aid within a clinical setting.

## Supplementary information

**Additional file 1.** Topic Guide: Anti-psychotic decision aid project: focus group topics of conversation and example of think aloud feedback.

**Additional file 2.** Your Medication Decision Aid.

## Data Availability

The authors are happy to respond to any reasonable requests for data.

## Abbreviations

ICD-10: 10th revision of the International Statistical Classification of Diseases and Related Health Problems, a medical classification list by the World Health Organisation
NHS: National Health Service, United Kingdom
QSR NVivo: Qualitative data analysis software package produced by QSR International
REC: Research Ethics Committee
WHO: World Health Organisation
