# Supplementary material for: Making decisions about antipsychotics: a qualitative study of patient experience and the development of a decision aid
Source: BMC Psychiatry. 2019 Oct 23;19:309. doi: 10.1186/s12888-019-2304-3 (PMC6806500; doi:10.1186/s12888-019-2304-3)
Supplement: Supplementary file 1 — Additional file 1. Topic Guide: Anti-psychotic decision aid project: focus group topics of conversation and example of think aloud feedback. [file 12888_2019_2304_MOESM1_ESM.docx]

**Supplementary Materials**

Topic Guide: Anti-psychotic decision aid project: focus group topics of conversation

Introduction:

Aim of the group

Explain health and safety procedures, confidentiality and privacy considerations – e.g. first names only, no personal identifiable details, no “naming and shaming”.

Check if everyone has read through the information sheet, an opportunity to clarify any outstanding queries and have completed informed consent forms.

Remind all of rights to withdraw at any time before, during or after the group.

Remind all of support resources for anyone who may experience distress or simply want to talk about their experiences of participation following the group.

Group warm up exercise:

Ask participants to think about their previous experiences with medication and to write something down on the post it notes provided – post notes on the wall and open up to group discussion.

Example questions:

Thinking back on the antipsychotic medication you have received in the past, what comes to mind?

Is there anything you wish you knew about an antipsychotic medication before you started taking it?

Are there any particular experiences with medication that stay with you?

Discussion:

Desired benefits from taking an antipsychotic medication:

In an ideal world what would you like an antipsychotic medication to do?

What is it about your experiences that you would most like a medication to help with?

Side effects from antipsychotic medication:

What side effects would you want to avoid?

What are the side-effects that you dislike the most and why?

Does your medication affect you in any negative way? Does it bother you? Why?

Route of administration:

Does the way the medication is given to you matter? E.g. tablet, dissolvable under the tongue tablet, depot injection. Why?

Do you struggle to remember taking tablets?

Have you ever been prescribed a long term antipsychotic injection? What was it like? Why?

Monitoring:

How do you feel about the need for blood tests and other physical tests like heart monitoring before/when you are taking a medication?

Decision making:

What information do you use to make decisions about which antipsychotic medication you take?

Where do you get information to make decisions about your medication? E.g. do you look online? Do you speak with your psychiatrist? Do you speak to other people with psychosis?

What would help you to make better decisions about your medication?

Do you feel you have enough time to make informed decisions about your medication?

Do you think the information accessible to you is easy enough to understand?

How easy is it to question your psychiatrist? What was the last thing you asked your psychiatrist? What would make it easier to ask your psychiatrist a question?

What would help you to decide to keep taking the medication as prescribed for a year, two years or the rest of your life?

Example of think aloud feedback

P001

• [title]

To be honest I don’t understand….{reading}….your medication…. Its something to help you…

You gotta make it simpler than that

• [medication history]

… about my medication if I can remember it

…. Let them know where it is, I would never think it was at the back

R: we could have a list that you could tick…

P: yeah that would be more easier

[Section 3]

• {reading} … what would you like your medication to help you with? ..Everything

• P: Put a tick thing there as well and they would know

R: so a visual thing helps

P: yeah

• P:…for me important, very important

R: and what about the rest of these ones

P: umm {reading}…. very important again for that…. {reading } that’s very important as well…. {reading}…. that very important…. {reading}…. all of that <gesturing to all of the above>

• P: That’s a good one aswell….…. {reading}….that’s a good one… that’s another one… that’s important aswell

• R: would you say are you putting very important for all of these

P: near enough, I could read it over again but the majority of this is important or very important… its not moderate important but I might come to that… umm I think... this way is better…sometimes with the scale thing…. Its not as easy too…umm…It’s not as easy as this

• {reading}Reduction in unusual experience…. What mean by that?

• R: do you this a scale would be better? Have like a line and you just have to mark on it?

P: on and tick it. I have had that before. I did that with Tracy before

• I don’t even have a computer …. I don’t know nothing about it, some people are so forward they would be happy with it on the phone

• If you got help, it shouldn’t take too long

P002

• [medication history]

“I would be confused ‘cause I can’t remember…I am on the clozapine…I can’t remember the other ones”

• [Section 6]

“ I don’t understand what this is saying …{researcher explains} …oh ”

• [Summary priority section]

R: Is that clear?

P: When everything is explained to you”

• [Further information links]

R: would you find that useful?

P: No I wouldn’t find that useful, I don’t use a computer much

• [Commenting over all]

P003

Audio file corrupted so unable to transcribe
