# Supplementary material for: Making decisions about antipsychotics: a qualitative study of patient experience and the development of a decision aid
Source: BMC Psychiatry. 2019 Oct 23;19:309. doi: 10.1186/s12888-019-2304-3 (PMC6806500; doi:10.1186/s12888-019-2304-3)
Supplement: Supplementary file 2 — Additional file 2. Your Medication Decision Aid. [file 12888_2019_2304_MOESM2_ESM.docx]

**Your**

**Medication Decision Aid**

**
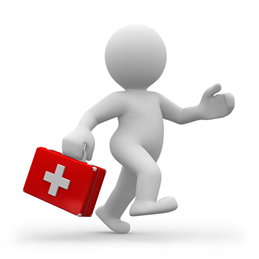
**

***Please complete and bring to your next meeting with your Doctor***

Helping you make decisions about your mental health medication

**Section 1: Introduction**

**Who is this decision aid for?**

People who are thinking about taking or changing medication.

**What is it?**

This decision aid is a way of **helping you** think about medication treatment. It will **help you** **make your wishes known when making a medication decision** with your doctor.

**How will it help?**

The decision aid should **help you to set out your thoughts** in a clear and easy to remember way to help you discuss medication choices with your doctor.

**How does a decision aid work?**

You will **write down your thoughts on the medications** you have taken in the past. This will help you to identify the **most important aspects of treatment for you**. At the end, the aid will ask you to summarise this information to help you decide about future medication when you next meet your doctor.

Please write your thoughts in the dotted boxes like this:

Olanzapine was helpful in reducing voices

**What should you do with the results?**

Once you have completed the decision aid, **take it to your next meeting** with your doctor or nurse so you can use it **to help discuss medication options** with them. You could also discuss the decision aid with your family, friends, carers or anyone else you think could be helpful.

**Section 2: What are my experiences of medication?**

| 🞎 | Amisupride | 🞎 | Perphenazine |
| --- | --- | --- | --- |
| 🞎 | Aripiprazole | 🞎 | Paliperidone tablet |
| 🞎 | Asenapine | 🞎 | Pericyazine |
| 🞎 | Benperidol | 🞎 | Pimozide (Orap™) |
| 🞎 | Chlorpromazine (Largactil™) | 🞎 | Pipotiazine (Piportil™) |
| 🞎 | Clozapine | 🞎 | Promazine |
| 🞎 | Flupentixol (Depixol™) | 🞎 | Promethiazine |
| 🞎 | Fluphenazine (Modecate™) | 🞎 | Quetiapine |
| 🞎 | Haloperidol tablet | 🞎 | Risperidone |
| 🞎 | Haloperidol depot injection (Haldol™) | 🞎 | Risperidone injection (Risperdal™) |
| 🞎 | Levomepromazine | 🞎 | Sulpiride Trifluoperazine (Stelazine™) |
| 🞎 | Olanzapine tablet | 🞎 | Zuclopenthixol (Clopixol™) |
| 🞎 | Olanzapine injection | 🞎 | Paliperidone injection |

Please Tick “**✔** ” all the medications you have had in the past

**If your medication is not on this list, please write it on line below**

**The medication I found the most helpful was:**

Why?

**The medication I found the least helpful was:**

Why?

**Section 3: What would you like the medication to help with?**

**The most troubling aspect of my mental health problem in the last month has been:**

**It is very important we know what you think about your treatment. Please help us by completing answering the questions below.**

**Mark your answers by putting a “✔”in the relevant box. Ensure you select only one answer per question.**

**How important to you is:**

**A reduction in hearing voices:**

| **Most**  **Important** | **Very Important** | **Kind of Important** | **Doesn’t Bother me** | **Not Important** |  | **Does Not Apply** |
| --- | --- | --- | --- | --- | --- | --- |
| **🞎** | **🞎** | **🞎** | **🞎** | **🞎** |  | **🞎** |

**A reduction in seeing unusual things:**

| **Most**  **Important** | **Very Important** | **Kind of Important** | **Doesn’t Bother me** | **Not Important** |  | **Does Not Apply** |
| --- | --- | --- | --- | --- | --- | --- |
| **🞎** | **🞎** | **🞎** | **🞎** | **🞎** |  | **🞎** |

**Feeling less paranoid:**

| **Most**  **Important** | **Very Important** | **Kind of Important** | **Doesn’t Bother me** | **Not Important** |  | **Does Not Apply** |
| --- | --- | --- | --- | --- | --- | --- |
| **🞎** | **🞎** | **🞎** | **🞎** | **🞎** |  | **🞎** |

**Being able to concentrate better:**

| **Most**  **Important** | **Very Important** | **Kind of Important** | **Doesn’t Bother me** | **Not Important** |  | **Does Not Apply** |
| --- | --- | --- | --- | --- | --- | --- |
| **🞎** | **🞎** | **🞎** | **🞎** | **🞎** |  | **🞎** |

**Being able to return to education/work:**

| **Most**  **Important** | **Very Important** | **Kind of Important** | **Doesn’t Bother me** | **Not Important** |  | **Does Not Apply** |
| --- | --- | --- | --- | --- | --- | --- |
| **🞎** | **🞎** | **🞎** | **🞎** | **🞎** |  | **🞎** |

**Sleeping better:**

| **Most**  **Important** | **Very Important** | **Kind of Important** | **Doesn’t Bother me** | **Not Important** |  | **Does Not Apply** |
| --- | --- | --- | --- | --- | --- | --- |
| **🞎** | **🞎** | **🞎** | **🞎** | **🞎** |  | **🞎** |

**Thinking more clearly:**

| **Most**  **Important** | **Very Important** | **Kind of Important** | **Doesn’t Bother me** | **Not Important** |  | **Does Not Apply** |
| --- | --- | --- | --- | --- | --- | --- |
| **🞎** | **🞎** | **🞎** | **🞎** | **🞎** |  | **🞎** |

**Feeling less worried:**

| **Most**  **Important** | **Very Important** | **Kind of Important** | **Doesn’t Bother me** | **Not Important** |  | **Does Not Apply** |
| --- | --- | --- | --- | --- | --- | --- |
| **🞎** | **🞎** | **🞎** | **🞎** | **🞎** |  | **🞎** |

**Getting on better with people:**

| **Most**  **Important** | **Very Important** | **Kind of Important** | **Doesn’t Bother me** | **Not Important** |  | **Does Not Apply** |
| --- | --- | --- | --- | --- | --- | --- |
| **🞎** | **🞎** | **🞎** | **🞎** | **🞎** |  | **🞎** |

**Staying out of hospital:**

| **Most**  **Important** | **Very Important** | **Kind of Important** | **Doesn’t Bother me** | **Not Important** |  | **Does Not Apply** |
| --- | --- | --- | --- | --- | --- | --- |
| **🞎** | **🞎** | **🞎** | **🞎** | **🞎** |  | **🞎** |

**Not having my thoughts or feelings interfered with:**

| **Most**  **Important** | **Very Important** | **Kind of Important** | **Doesn’t Bother me** | **Not Important** |  | **Does Not Apply** |
| --- | --- | --- | --- | --- | --- | --- |
| **🞎** | **🞎** | **🞎** | **🞎** | **🞎** |  | **🞎** |

**A reduction in unusual experiences:**

| **Most**  **Important** | **Very Important** | **Kind of Important** | **Doesn’t Bother me** | **Not Important** |  | **Does Not Apply** |
| --- | --- | --- | --- | --- | --- | --- |
| **🞎** | **🞎** | **🞎** | **🞎** | **🞎** |  | **🞎** |

**Feeling less depressed or irritable:**

| **Most**  **Important** | **Very Important** | **Kind of Important** | **Doesn’t Bother me** | **Not Important** |  | **Does Not Apply** |
| --- | --- | --- | --- | --- | --- | --- |
| **🞎** | **🞎** | **🞎** | **🞎** | **🞎** |  | **🞎** |

**Having more stable moods:**

| **Most**  **Important** | **Very Important** | **Kind of Important** | **Doesn’t Bother me** | **Not Important** |  | **Does Not Apply** |
| --- | --- | --- | --- | --- | --- | --- |
| **🞎** | **🞎** | **🞎** | **🞎** | **🞎** |  | **🞎** |

**If there is anything else, please write it on the line below:**

**Section 3: Positive effects of medication summary**


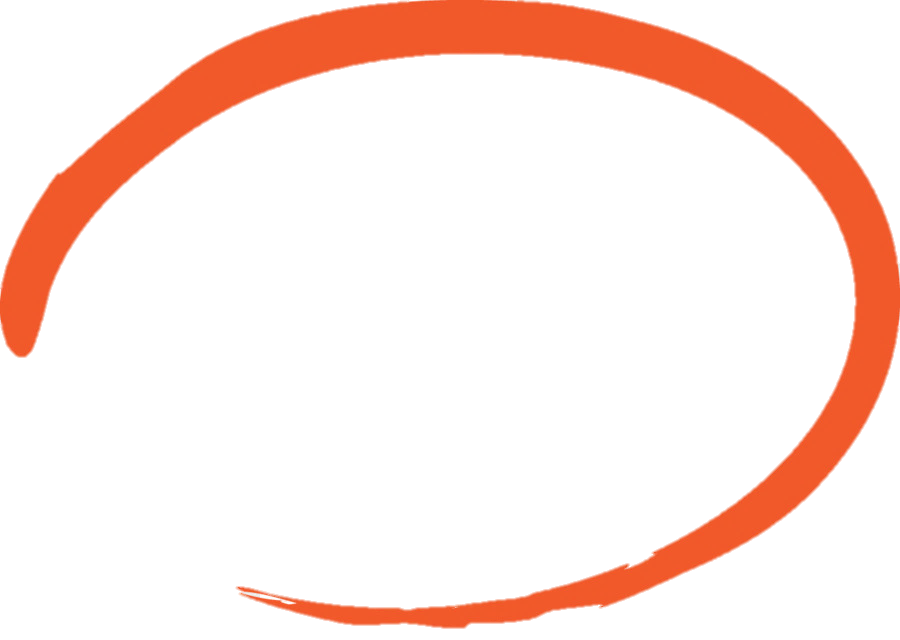
**Please review your previous responses and then circle ” “ the 3 most important things you want medication to help you with:**

| Thoughts Interfered With | | Concentration | | Stable Mood | |
| --- | --- | --- | --- | --- | --- |
| Paranoia | | Depressed /  Irritable | | Education / Work | |
| Sleep | | Clear Thinking | | Less Worried | |
| Unusual Experiences | | Staying out of Hospital | | Hearing Voices | |
|  | Seeing Things | | Get along with People | |  |

**To me, the most important positive effect of treatment would be:**

**Section 4: How do I feel about possible side-effects?**

**Please consider that most medications can cause some temporary side effects**

**Mark your answers by putting a “✔”in the relevant box.**

**Experiencing a tremor (Shaking):**

| **Could NOT Cope with** | **Would Bother me** | **Could Cope with** | **Would NOT Bother Me** |  | **Does Not Apply** |
| --- | --- | --- | --- | --- | --- |
| **🞎** | **🞎** | **🞎** | **🞎** |  | **🞎** |

**Feeling tired:**

| **Could NOT Cope with** | **Would Bother me** | **Could Cope with** | **Would NOT Bother Me** |  | **Does Not Apply** |
| --- | --- | --- | --- | --- | --- |
| **🞎** | **🞎** | **🞎** | **🞎** |  | **🞎** |

**Sexual problems:**

| **Could NOT Cope with** | **Would Bother me** | **Could Cope with** | **Would NOT Bother Me** |  | **Does Not Apply** |
| --- | --- | --- | --- | --- | --- |
| **🞎** | **🞎** | **🞎** | **🞎** |  | **🞎** |

**Experiencing breast enlargement or producing breast milk:**

| **Could NOT Cope with** | **Would Bother me** | **Could Cope with** | **Would NOT Bother Me** |  | **Does Not Apply** |
| --- | --- | --- | --- | --- | --- |
| **🞎** | **🞎** | **🞎** | **🞎** |  | **🞎** |

**Gaining weight:**

| **Could NOT Cope with** | **Would Bother me** | **Could Cope with** | **Would NOT Bother Me** |  | **Does Not Apply** |
| --- | --- | --- | --- | --- | --- |
| **🞎** | **🞎** | **🞎** | **🞎** |  | **🞎** |

**Having stiff muscles:**

| **Could NOT Cope with** | **Would Bother me** | **Could Cope with** | **Would NOT Bother Me** |  | **Does Not Apply** |
| --- | --- | --- | --- | --- | --- |
| **🞎** | **🞎** | **🞎** | **🞎** |  | **🞎** |

**Having a dry mouth:**

| **Could NOT Cope with** | **Would Bother me** | **Could Cope with** | **Would NOT Bother Me** |  | **Does Not Apply** |
| --- | --- | --- | --- | --- | --- |
| **🞎** | **🞎** | **🞎** | **🞎** |  | **🞎** |

**Having a lot of saliva:**

| **Could NOT Cope with** | **Would Bother me** | **Could Cope with** | **Would NOT Bother Me** |  | **Does Not Apply** |
| --- | --- | --- | --- | --- | --- |
| **🞎** | **🞎** | **🞎** | **🞎** |  | **🞎** |

**Being sensitive to the sun:**

| **Could NOT Cope with** | **Would Bother me** | **Could Cope with** | **Would NOT Bother Me** |  | **Does Not Apply** |
| --- | --- | --- | --- | --- | --- |
| **🞎** | **🞎** | **🞎** | **🞎** |  | **🞎** |

**Developing diabetes:**

| **Could NOT Cope with** | **Would Bother me** | **Could Cope with** | **Would NOT Bother Me** |  | **Does Not Apply** |
| --- | --- | --- | --- | --- | --- |
| **🞎** | **🞎** | **🞎** | **🞎** |  | **🞎** |

**Developing high cholesterol:**

| **Could NOT Cope with** | **Would Bother me** | **Could Cope with** | **Would NOT Bother Me** |  | **Does Not Apply** |
| --- | --- | --- | --- | --- | --- |
| **🞎** | **🞎** | **🞎** | **🞎** |  | **🞎** |

**Having blurred vision:**

| **Could NOT Cope with** | **Would Bother me** | **Could Cope with** | **Would NOT Bother Me** |  | **Does Not Apply** |
| --- | --- | --- | --- | --- | --- |
| **🞎** | **🞎** | **🞎** | **🞎** |  | **🞎** |

**Having a fast heart beat:**

| **Could NOT Cope with** | **Would Bother me** | **Could Cope with** | **Would NOT Bother Me** |  | **Does Not Apply** |
| --- | --- | --- | --- | --- | --- |
| **🞎** | **🞎** | **🞎** | **🞎** |  | **🞎** |

**Having constipation:**

| **Could NOT Cope with** | **Would Bother me** | **Could Cope with** | **Would NOT Bother Me** |  | **Does Not Apply** |
| --- | --- | --- | --- | --- | --- |
| **🞎** | **🞎** | **🞎** | **🞎** |  | **🞎** |

**Soreness at the site of an injection:**

| **Could NOT Cope with** | **Would Bother me** | **Could Cope with** | **Would NOT Bother Me** |  | **Does Not Apply** |
| --- | --- | --- | --- | --- | --- |
| **🞎** | **🞎** | **🞎** | **🞎** |  | **🞎** |

**If there is anything else, please write it on the line below and tick to show how important it is:**

| **Could NOT Cope with** | **Would Bother me** | **Could Cope with** | **Would NOT Bother Me** |  | **Does Not Apply** |
| --- | --- | --- | --- | --- | --- |
| **🞎** | **🞎** | **🞎** | **🞎** |  | **🞎** |

**Section 4: Side effects summary**

**To me the most important potential side effects to avoid would be:**

**Section 5: How do I feel about blood tests and other physical health checks?**

**Having a bloods test to check my physical health is something I would like to ...**

| **Avoid at all Costs** | **Avoid as much as Possible** | **Do Not Mind** | **Think is Important** | **Think is Very Important** |
| --- | --- | --- | --- | --- |
| **🞎** | **🞎** | **🞎** | **🞎** | **🞎** |

**Having regular heart tracings is something I would…**

| **Avoid at all Costs** | **Avoid as much as Possible** | **Do Not Mind** | **Think is Important** | **Think is Very Important** |
| --- | --- | --- | --- | --- |
| **🞎** | **🞎** | **🞎** | **🞎** | **🞎** |

**To me the most important aspects of physical health checks when taking medication are:**

**Section 6: How would I like to take medication?**

**Introduction:**

Medication can be given in three main ways:

1. A tablet that you take one or more times everyday

2. A tablet that you dissolve under your tongue one or more times a day

3. An injection given every two weeks to 3 months

**Please select how much you agree with the following statements:**

**I sometimes forget my medication**

| **Strongly Disagree** | **Disagree** | **Slightly Disagree** | **Undecided** | | **Slightly Agree** | **Agree** | **Strongly Agree** |
| --- | --- | --- | --- | --- | --- | --- | --- |
| **🞎** | **🞎** | **🞎** | **🞎** | **🞎** | | **🞎** | **🞎** |

**I would like something that is easy to remember**

| **Strongly Disagree** | **Disagree** | **Slightly Disagree** | **Undecided** | | **Slightly Agree** | **Agree** | **Strongly Agree** |
| --- | --- | --- | --- | --- | --- | --- | --- |
| **🞎** | **🞎** | **🞎** | **🞎** | **🞎** | | **🞎** | **🞎** |

**I would like something I could take once very few weeks**

| **Strongly Disagree** | **Disagree** | **Slightly Disagree** | **Undecided** | | **Slightly Agree** | **Agree** | **Strongly Agree** |
| --- | --- | --- | --- | --- | --- | --- | --- |
| **🞎** | **🞎** | **🞎** | **🞎** | **🞎** | | **🞎** | **🞎** |

**I prefer control of when I take my medication**

| **Strongly Disagree** | **Disagree** | **Slightly Disagree** | **Undecided** | | **Slightly Agree** | **Agree** | **Strongly Agree** |
| --- | --- | --- | --- | --- | --- | --- | --- |
| **🞎** | **🞎** | **🞎** | **🞎** | **🞎** | | **🞎** | **🞎** |

**I prefer not to have to think about taking medication**

| **Strongly Disagree** | **Disagree** | **Slightly Disagree** | **Undecided** | | **Slightly Agree** | **Agree** | **Strongly Agree** |
| --- | --- | --- | --- | --- | --- | --- | --- |
| **🞎** | **🞎** | **🞎** | **🞎** | **🞎** | | **🞎** | **🞎** |

**I find swallowing tablets hard**

| **Strongly Disagree** | **Disagree** | **Slightly Disagree** | **Undecided** | | **Slightly Agree** | **Agree** | **Strongly Agree** |
| --- | --- | --- | --- | --- | --- | --- | --- |
| **🞎** | **🞎** | **🞎** | **🞎** | **🞎** | | **🞎** | **🞎** |

**I would consider having medication as** **an injection:**

| **Strongly Disagree** | **Disagree** | **Slightly Disagree** | **Undecided** | | **Slightly Agree** | **Agree** | **Strongly Agree** |
| --- | --- | --- | --- | --- | --- | --- | --- |
| **🞎** | **🞎** | **🞎** | **🞎** | **🞎** | | **🞎** | **🞎** |

**Section 6: How a medication is taken summary**

**To me the most important thing about how medication is taken is:**

**
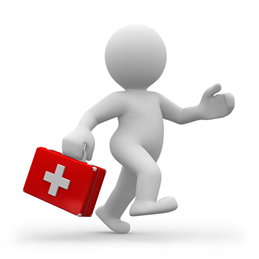
**Nearly finished!

**Section 7: Decision aid**

**summary**

**Considering all the things related to medication that you have thought about, which 3 are the most important to you (1 – most important, 3 – least important)?**

**My top 3 most important things to consider when choosing medication are:**

1.

2.

3.

**Are there any comments or feedback you want to give us?**
